# A Case of Docetaxel-Induced Left Ventricular Outflow Tract Obstruction

**DOI:** 10.7759/cureus.43598

**Published:** 2023-08-16

**Authors:** Taiga Mase, Sakiko Honda, Michiyo Yamano, Tatsuya Kawasaki

**Affiliations:** 1 Department of Cardiology, Matsushita Memorial Hospital, Moriguchi, JPN

**Keywords:** left ventricular outflow tract, high-output, heart failure, fluid retention, docetaxel

## Abstract

Docetaxel, a taxoid chemotherapy agent, may induce fluid retention. We present a case of metastatic breast cancer in which high output caused by docetaxel-induced fluid retention resulted in heart failure due to left ventricular outflow tract (LVOT) obstruction. A 58-year-old woman presented with exertional dyspnea and anasarca. The jugular venous pressure was elevated, and the carotid pulse was pulsus bisferiens with a spike-and-dome configuration. On auscultation, a mid-late systolic murmur that did not radiate to the neck but increased with the Valsalva maneuver was noted. Echocardiography revealed a left ventricular ejection fraction of 63% with systolic anterior motion (SAM) of the mitral valve, resulting in LVOT obstruction with a resting pressure gradient of 64 mmHg and moderate to severe mitral regurgitation. Treatment with carvedilol, trichlormethiazide, and an increased dose of furosemide gradually improved her symptoms, physical findings, and echocardiographic abnormalities. This case highlights the importance of recognizing high-output heart failure along with LVOT obstruction in patients scheduled to receive docetaxel.

## Introduction

Docetaxel, a semisynthetic taxoid, promotes microtubule polymerization and inhibits tubulin depolymerization, resulting in the inability of cells to replicate [1]. More recently, docetaxel has been widely used in the treatment of various cancers, including breast cancer, but attention should be paid to fluid retention as a unique side effect [1,2]. Here, we report a case of metastatic breast cancer in which high output by fluid retention during treatment with docetaxel resulted in heart failure due to left ventricular outflow tract (LVOT) obstruction.

## Case presentation

A 58-year-old woman was admitted because of exertional dyspnea and anasarca. She had been in her normal state of health until several months before presentation when anasarca had developed gradually after the administration of docetaxel for the treatment of breast cancer with bone metastases. The patient reported that she had gained approximately 6 kilograms of weight over the past four months. The last administration of docetaxel in combination with dexamethasone was two weeks before this admission, and the total dose of docetaxel was 780 mg. Approximately one week before admission, she had noticed dyspnea on exertion, and the symptom had gradually worsened, along with a weight gain of an additional 6 kilograms in the past two weeks. Her medical history was otherwise notable for osteoarthritis of the knees. Her medications included furosemide 20 mg daily, tramadol hydrochloride 37.5 mg daily, and acetaminophen 325 mg as needed for pain. She did not drink or smoke and had no known allergies. There was no family history of cardiovascular disease including hypertrophic cardiomyopathy.

On examination, she was lethargic. Her blood pressure was 124/67 mmHg, her pulse rate was 117 beats per minute, and her oxygen saturation level was 95% while she was breathing ambient air. The jugular venous pulsation was elevated to 18 cmH_2_O, and the carotid pulse was notable for pulsus bisferiens with a spike-and-dome configuration. Chest auscultation revealed a mid-late systolic murmur (grade 3/6) that did not radiate to the neck and increased with the Valsalva maneuver or from sitting to standing. Breath sounds were decreased in the bilateral lower lung fields, and there was severe pitting edema in the bilateral legs.

Electrocardiography showed a heart rate of 116 beats per minute and a QS pattern in leads V_1_ to V_3_ without ST-T segment changes. A chest radiograph showed cardiomegaly, pulmonary congestion, and bilateral pleural effusions. Her complete blood cell count was normal except for a hemoglobin of 10.2 g/dl, which was unchanged from two weeks earlier. Her electrolyte balance was normal, as were liver and thyroid function tests. The levels of C-reactive protein, urea nitrogen, and creatinine were 0.57 mg/dl, 25 mg/dl, and 0.93 mg/dl, respectively, findings unchanged from the previous data obtained two weeks earlier. The brain natriuretic peptide level was 23.0 pg/ml (reference value ≤18.4), and the high sensitivity troponin T level was 0.082 ng/ml (reference value ≤0.014).

**Figure 1:**
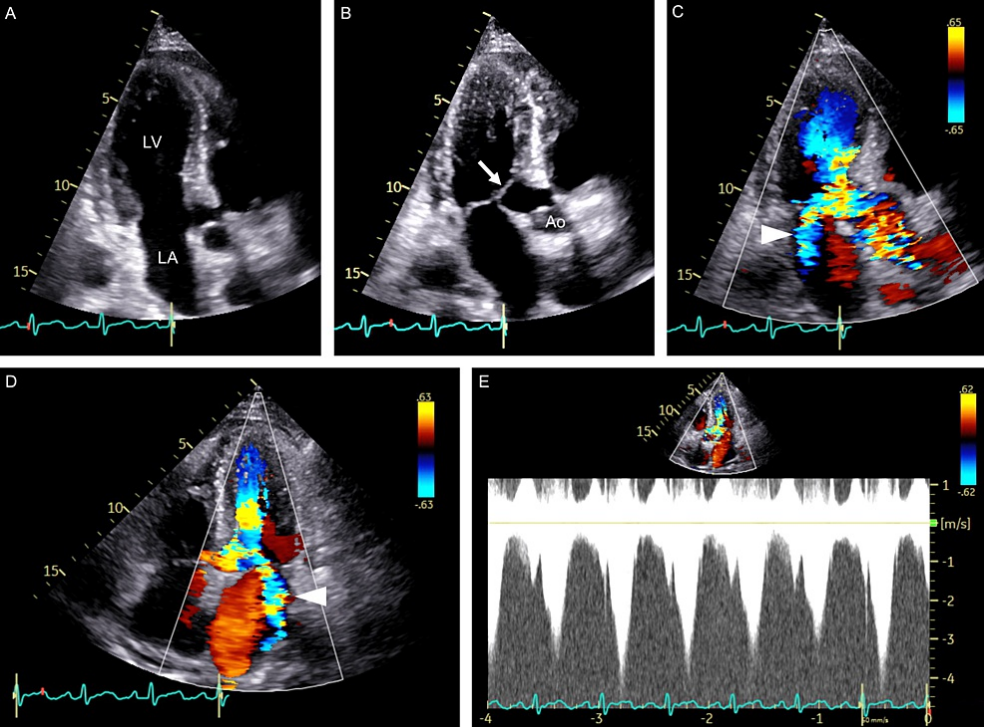
Echocardiography Apical three-chamber views at end-diastole (A) and end-systole (B) show SAM of the mitral valve (arrow). Color Doppler images of an apical three-chamber view (C) and four-chamber view (D) show an LVOT obstruction, along with mitral regurgitation (arrowheads), with a peak flow velocity of 4.0 m/s (E) Ao: aorta, LA: left atrium, LV: left ventricle

Echocardiography revealed a left ventricular ejection fraction of 63% with no wall motion abnormalities in either ventricle. Of note, systolic anterior motion (SAM) of the mitral valve was demonstrated, resulting in LVOT obstruction with a resting pressure gradient of 64 mmHg accompanied by moderate to severe mitral regurgitation (Figure 1), none of which had been observed 10 months earlier except for SAM without septal contact. No findings suggestive of hypertrophic cardiomyopathy were detected, although mild hypertrophy was observed in the left ventricle, e.g., an interventricular septal thickness of 13 mm. Doppler imaging showed a mitral valve E of 0.64 m/s, a decorrelation time of the E wave of 111 ms, an E/A ratio of 0.75, a septal mitral annular early peak velocity (e') of 6.0, and an E/e’ ratio of 10.7. The cardiac output and cardiac index were calculated to be 9.8 l/min and 6.1 l/min/m^2^, respectively. The systolic pulmonary blood pressure was estimated to be 48 mmHg. A diagnosis of high-output heart failure with LVOT obstruction was made. Cardiac magnetic resonance did not reveal findings consistent with hypertrophic cardiomyopathy, and genetic testing was not performed.

Additional history-taking revealed that the patients had been eating almost only white rice and noodles for approximately a month before the current admission, and a working diagnosis of cardiac beriberi or high-output heart failure due to thiamine deficiency was made. Vitamin B1 was added for a few days, but the cardiac output as assessed by echocardiography did not decrease. The thiamine level was later confirmed to be 28 ng/ml (reference value, 24-66). Her symptoms, physical findings including anasarca, and echocardiographic abnormalities had gradually improved with treatment with carvedilol, trichlormethiazide, and an increased dose of furosemide.

Three weeks after admission, she was discharged home in stable condition. Follow-up echocardiography performed two months after discharge showed an output of 3.2 l/min with no evidence of LVOT obstruction at rest and with the Valsalva maneuver, although SAM without septal contact was still present. Treatment for breast cancer is being considered without the use of docetaxel.

## Discussion

Our patient developed anasarca after receiving docetaxel for the treatment of metastatic breast cancer. Given the high incidence of fluid retention associated with this drug [1-3], it is reasonable to consider that the anasarca was attributed to docetaxel. The patient also developed high-output heart failure with LVOT obstruction, which gradually decreased and eventually disappeared in accordance with the improvement of high output. Although the underlying mechanism linking LVOT obstruction and high output remains unclear, it is safe to assume that docetaxel-induced fluid retention provoked high-output heart failure with LVOT obstruction in this patient because SAM without septal contact can progress to LVOT obstruction due to hyperkinetic wall motion.

Similar to other chemotherapeutic agents, the side effects of docetaxel include neutropenia, alopecia, myalgia, mucositis, neuropathy, hypersensitivity reactions, nail changes, and skin reactions [2]. It is also important to note that this drug causes fluid retention, such as swelling of the extremities, pleural and pericardial effusion, ascites, and anasarca; dose-cumulative severe fluid retention has been reported in 6.5% of docetaxel recipients [2]. The exact mechanism underlying docetaxel-induced fluid retention remains unclear, but increased capillary permeability may cause leakage of fluid into the surrounding tissues [4]. Corticosteroids, even a single dose of dexamethasone, have been reported to have a beneficial effect on docetaxel-induced fluid retention [5,6], but in the current patient, anasarca developed despite receiving multiple doses of dexamethasone.

Docetaxel exerts its cytotoxic effect by enhancing microtubule assembly and stabilizing microtubules [1], resulting in increased microtubules that may induce contractile dysfunction [7]. In this case, contractile dysfunction was an unlikely cause of heart failure as the wall motion of both ventricles was preserved in a series of echocardiographic studies. It is reported that docetaxel can induce left ventricular diastolic dysfunction as assessed by echocardiographic variables such as mitral inflow E/A ratio and deceleration time and brain natriuretic peptide levels [8], none of which were remarkable in our patient. The most notable cardiac finding in our case was high output up to approximately 10 l/min or 6 l/min/m^2^, which deserves attention as an unusual cause of heart failure.

High-output heart failure (e.g., defined as an elevated cardiac index of ≥4 l/min/m^2^ [9]) has a variety of etiologies, including chronic anemia, arteriovenous shunts, hyperthyroidism, obesity, liver disease, lung disease, thiamine deficiency, and physiologic (e.g., pregnancy, fever, infection) or iatrogenic conditions (e.g., pulmonary vasodilators, inotropes) [9,10], none of which were found in this case. Thus, it is reasonable to consider docetaxel-induced fluid retention as the cause of the high output, although the possibility of the effect of thiamine on the development of the high output cannot be ruled out since the blood level of thiamine was close to the lower limit of the normal range.

Fluid accumulation in the body can increase the volume of blood in the circulatory system, leading to an increase in cardiac output. LVOT obstruction has been reported to develop not only in hypertrophic obstructive cardiomyopathy but also in other conditions such as hypertensive hypertrophy, sigmoid septum, and hyperkinetic left ventricle [11]. Not surprisingly, increased cardiac workload due to docetaxel-induced fluid retention provokes LVOT obstruction in patients with SAM without septal contact. A treatment dilemma exists when LVOT obstruction and high cardiac workload coexist, as in our case, because fluid infusion or negative inotropic agents may worsen heart failure even though these treatments result in relief of LVOT obstruction. In this case, careful treatment with beta-blockers and diuretics relieved her symptoms and LVOT obstruction as the cardiac output returned to normal levels. Further research is needed to examine the potential long-term effects of docetaxel treatment on cardiac function and the risk of developing LVOT obstruction and high-output heart failure, as demonstrated in the current patient.

## Conclusions

We experienced a case of high-output heart failure with LVOT obstruction due to docetaxel-induced fluid retention. The current case highlights the importance of recognizing SAM without septal contact as a possible trigger of heart failure due to LVOT obstruction in a patient scheduled to receive docetaxel, even in the absence of risk factors for cardiovascular disease.
